# Comparing the effectiveness of emotion regulation therapy and cognitive behavioral therapy on treatment adherence in hemodialysis patients: A randomized controlled clinical trial

**DOI:** 10.1371/journal.pone.0339162

**Published:** 2025-12-26

**Authors:** Seyedeh Hanieh Salimi Arshad Moghaddam Pishkhani, Mohammad Javad Tarrahi, Fatemeh Zargar

**Affiliations:** 1 Department of Health Psychology, School of Medicine, Isfahan University of Medical Sciences, Isfahan, Iran; 2 Department of Epidemiology and Biostatistics, School of Health, Isfahan University of Medical Sciences, Isfahan, Iran; 3 Department of Health Psychology, Isfahan University of Medical Sciences, Isfahan, Iran; Warren Alpert Medical School of Brown University: Brown University Warren Alpert Medical School, UNITED STATES OF AMERICA

## Abstract

**Introduction:**

Non-adherence is a common challenge among patients undergoing hemodialysis (HD). This randomized controlled trial compared the efficacy of Emotion Regulation Therapy (ERT) and Cognitive Behavioral Therapy (CBT) on improving treatment adherence in hemodialysis patients, with a control group receiving standard care.

**Materials and methods:**

Ninety hemodialysis patients were equally randomized into ERT, CBT, and control groups (N = 30 each group), with six attrition cases per group. Baseline demographics (age, BMI, dialysis duration, education) showed no significant intergroup differences (p > 0.05). Adherence was assessed across five domains: dialysis, medication, fluid intake, dietary regimen, and total adherence. A MANCOVA/ANCOVA model analyzed changes at pre-intervention, post-intervention, and 3-month follow-up, controlling for baseline characteristics.

**Results:**

Both intervention groups demonstrated significant adherence improvements post-treatment versus controls (P < 0.001). CBT showed superior immediate effects, with total adherence scores increasing from 836.66 ± 192.95 to 1073.33 ± 89.28 (Δ + 28.3%), while ERT improved from 833.33 ± 210.53 to 920.00 ± 181.04 (Δ + 10.4%). At follow-up, CBT maintained higher adherence (1050.83 ± 93.88 vs. ERT’s 890.00 ± 155.30), though both groups experienced dialysis adherence declines from post-treatment peaks (CBT: 580 to 574.16; ERT: 520 to 483.33). Control group adherence deteriorated across all domains (total: 911.66 to 835.00). Time-intervention interactions were significant for total adherence (P < 0.001), dialysis (P = 0.006), and medication adherence (P < 0.001), with largest CBT effects on fluid restriction adherence (Δ + 56.1% vs. ERT’s Δ + 8.3%).

**Discussion:**

While both therapies enhanced adherence, CBT produced greater short-term improvements, particularly in behavioral domains (fluid/dietary compliance), whereas ERT showed better maintenance of medication adherence. The differential trajectory patterns suggest CBT’s structured behavioral strategies may offer immediate benefits, while ERT’s emotion-focused techniques could support longer-term regimen acceptance. Integration of both approaches into renal care protocols may optimize adherence outcomes.

## 1. Introduction

Progressive chronic kidney disease (CKD) and subsequent kidney failure, traditionally termed end-stage kidney disease (ESKD), pose significant public health challenges, characterized by high rates of morbidity and mortality. The World Health Organization (WHO) approximates that roughly 10% of the world’s population, equating to about 850 million individuals, is affected by CKD [1]. Patients with end-stage renal disease (ESRD) a condition that can be fatal, require renal replacement therapy, such as dialysis or a kidney transplant [2]. More than 97% of individuals newly diagnosed with kidney failure rely on dialysis therapy for treatment [3]. In numerous countries worldwide, hemodialysis is the most commonly used modality of dialysis [4].

Patients receiving dialysis who have ESKD must make considerable lifestyle adjustments, which includes following special dietary and fluid restrictions, adhering to complex renal replacement therapy protocols, and taking multiple medications [5]. The typical treatment plan for CKD patients may involve hemodialysis, medication, and dietary guidelines that restrict fluid and sodium consumption [6]. Patients struggle to maintain a routine that requires strict control of fluid intake, diet, medications, and lengthy dialysis sessions [7]. While advancements in dialysis technology are improving patient life expectancy, the majority of patients undergoing treatment have the ESRD, and must also manage associated symptoms such as fatigue, insomnia, pain from an arteriovenous fistula puncture, anxiety, and pressure, must be managed [8–11].

Non-adherence is a common challenge among both hemodialysis (HD) and peritoneal dialysis (PD) patients [12]. The majority of studies in the literature concentrate on non-adherence in HD patients [13,14]. Depending on the definition of non-adherence, various studies have demonstrated that its rate among hemodialysis patients can reach 80% [15]. Non-adherence has several negative effects, including increased hospital admission rates, a greater financial burden, and higher mortality [16,17].

Treatment adherence reflects how well patients follow the medical plan they create with their healthcare providers. It is essentially a measure of how closely a patient’s actions align with the agreed-upon health advice [18]. Adherence to behavioral adaptations is essential for effective disease management, even when they are difficult and require continuous regulation. Numerous unfavorable clinical outcomes are linked to non-adherence [13].

One way to conceptualize non-adherence is as either intentional or unintentional [19,20]. The term “intentional noncompliance” describes a deliberate decision to refuse or disregard medical advice. Unintentionally defying a healthcare provider’s recommendation, such as by forgetting, is known as nonintentional non-adherence. It is believed that the majority of dialysis patients, whether intentionally or unintentionally, do not follow their dialysis therapy to the letter [21]. In fact, a lot of hemodialysis patients who have been on the treatment long term understand how much latitude they have regarding dietary and fluid limitations and adjust their behavior accordingly [21]. Adherence behaviors may provide psychological benefits by elevating self-esteem and enhancing perceptions of internal control and mastery [21].

Literature reviews demonstrate that cognitive and behavioral interventions, such as Mindfulness Based Stress Reduction (MBSR) and CBT, significantly improve patient adherence and psychological outcomes [22–26]. CBT, a structured, time-limited approach, focuses on present day challenges by addressing the interplay between thoughts, behaviors, and physiological responses [27]. Through techniques like education, cognitive restructuring, and behavioral experiments, CBT enhances treatment adherence a critical factor in managing ESRD [25,27–29]. Non-adherence to dietary restrictions, medications, or dialysis schedules in ESRD patients severely impacts health outcomes, underscoring the need for effective interventions.

While CBT has proven effective in improving adherence among hemodialysis patients by targeting maladaptive thoughts and behaviors, ERT has emerged as a promising alternative. ERT integrates traditional CBT with mindfulness and emotion-focused strategies to address motivational barriers and improve emotional regulation [30]. Although ERT shows potential in enhancing psychological well-being and adherence in chronic illnesses, direct comparisons between CBT and ERT for hemodialysis patients remain unexplored [30,31]. This gap is particularly relevant given that some patients, such as those with complex conditions like Generalized Anxiety Disorder (GAD), may not fully respond to standard CBT.

This study aimed to compare the efficacy of ERT and CBT in improving treatment adherence among chronic kidney disease patients undergoing hemodialysis. By evaluating these two evidence-based therapies, we sought to determine whether ERT’s focus on emotion regulation offers superior benefits over CBT’s cognitive-behavioral approach in this high need population.

## 2. Materials and methods

### 2.1. Study design

This clinical trial study was conducted between February, 20, 2023 and April, 17, 2024. Ninety-six hemodialysis patients were enrolled to undergo eight sessions of cognitive behavioral therapy, emotion regulation therapy and control group.

To minimize the effect of potential confounding variables, baseline demographic and clinical characteristics (age, gender, education, duration of dialysis, baseline adherence score) were collected and compared across the three groups. No statistically significant differences were found among the groups at baseline (P > 0.05), indicating successful randomization.

### 2.2. Sample size

This number of samples was taken from People who were referred to the hemodialysis department of receiving dialysis hospitals. The sample size was calculated using G*Power software (version 3.1.9.7) for a one-way ANOVA design. Moreover, the sample size for each experimental group was calculated using the following the Equation (1):


$$\begin{array}{c} n={(\lambda }_{g,\alpha ,\text{1}-\beta })/\Delta \;\;\text{and}\;\;\Delta =\frac{\text{1}}{\sigma^{\text{2}}}\;\sum_{i=\text{1}}^{k}{\left(\mu -\overset{―}{\mu }\right)}^{\text{2}} \end{array}$$ (1)

The parameters for this calculation were defined as follows:

Number of groups (*k*) = 3, Type I error (α) = 0.05, Type II error (*β*) = 0.2 (power = 80%); Group means: μ1 = 900, μ2 = 970, μ3 = 860; Pooled standard deviation (*σ*) = 130.26 (derived from adherence score variance) [32]; Grand mean (*μ*ˉ) = 910 (calculated as $\frac{\text{1}}{\text{3}}\sum_{i=\text{1}}^{\text{3}}\mu_{i}$).

Substituting these values into the equation yielded a required sample size of 30 participants per group. Consequently, the total sample size for the study was determined to be 90 participants, ensuring adequate statistical power to detect intergroup differences in adherence scores under the specified error thresholds. Randomization in this study was performed using the method of simple randomization. For this purpose, an online website (www.rresearchrandomizer.com) was used, and a list was generated in which the letter assigned to each number indicated the group to which the participant belonged.

### 2.3. Inclusion and exclusion criteria

The inclusion criteria for patient selection were: 1) age between 20 and 80 years; 2) Referral by a nephrologist with the diagnosis requiring dialysis; 3) A minimum level of education; 4) Completion of the informed consent form; 5) Absence of a serious psychiatric illness, such as major depression, bipolar disorder, or psychotic disorders, as assessed by a short diagnostic interview; 6) No history of substance abuse or addiction; 7) No participation in other psychotherapy programs within the preceding six months.

The exclusion criteria patients selection were: 1) Absence from more than two therapy sessions; 2) Occurrence of severe physical problems; 3) Hospitalization during the study period; 4) Failure to complete the questionnaire.

### 2.4. Procedure

**Fig 1:**
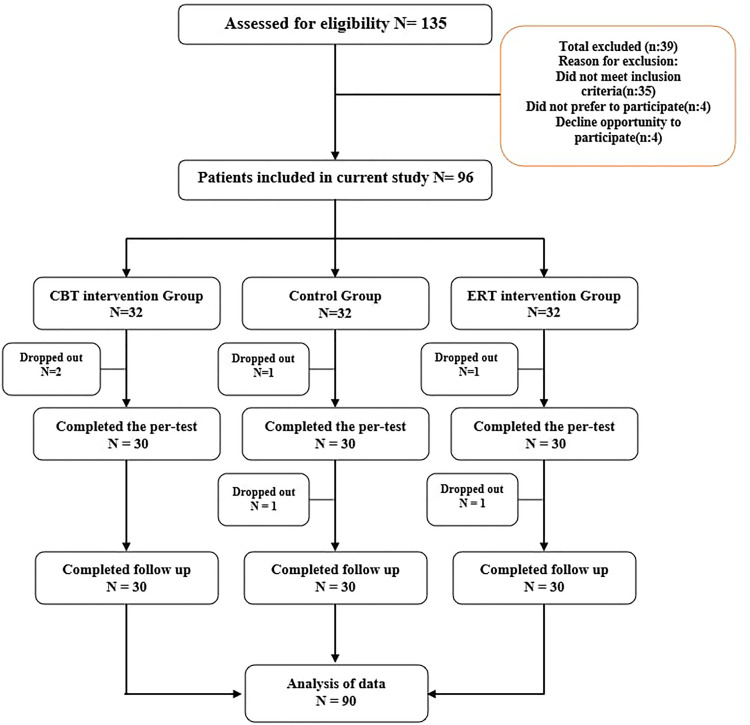
Flow diagram of study participants.

Participant enrollment started on the February, 20, 2023, and trial was completed on the April, 17, 2024, across different hospitals in Iran. Patients were approached in the hemodialysis centers and asked to undergo an assessment to determine their eligibility for the clinical trial. Following baseline assessment, eligible participants were randomly assigned to one of three groups: ERT, CBT, and control group. Pre-intervention assessments were performed. Subsequently, the two intervention groups received individual therapy for two months. After two months, post-intervention assessments were performed, and a three-month follow-up assessment was conducted. For the control group, the intervention was performed at the end of the study procedure. The following presents an overview of the study’s implementation framework (Fig 1).

### 2.5. Data collection tools

In this study, data were collected using a demographic questionnaire and ESRD-AQ questionnaire. The demographic questionnaire included items on age, gender, marital status, level of education, duration of hemodialysis, BMI, and the frequency of hemodialysis sessions per week.

#### 2.5.1. The end-stage renal disease adherence questionnaire (ESRD-AQ).

Treatment adherence was assessed using the ESRD-AQ, a validated instrument designed to measure adherence behaviors in patients receiving in center hemodialysis. The final version of the ESRD-AQ comprises 46 items, with its core focusing on four critical domains of adherence: hemodialysis attendance (14 items), medication use (9 items), fluid restrictions (10 items), and dietary recommendations (8 items). The scoring of the ESRD-AQ is methodologically rigorous and involves several steps to ensure clinical relevance and accuracy. The adherence behavior subscale, which forms the basis for the domain scores reported in this study, is calculated by summing the responses to key questions within each domain. Furthermore, a weighting system was applied to the scores based on the relative importance of each behavior to clinical outcomes; for instance, missing or shortening hemodialysis sessions) a behavior strongly linked to mortality (was assigned a greater weight. Additionally, the scores for specific questions concerning missed treatments (Questions 14 and 18) or medications (Question 26) were adjusted for medically justified reasons; patients providing a valid explanation (e.g., hemodialysis access problems) received a full score for those items. Consequently, the domain scores presented herein are weighted and adjusted composite scores, where higher values indicate superior adherence supporting information. The total adherence score was derived from the sum of these four domain score (S2 File) [33]. The Persian version of the ESRD-AQ employed in this research has demonstrated excellent content validity (CVI = 0.98) as confirmed by Rafiee Vardjani et al. [34].

### 2.6. Interventions

#### 2.6.1. Cognitive behavioral therapy.

Thirty-two patients received eight sessions of cognitive behavioral therapy. Participants in the CBT underwent eight weekly, 60-minute individual sessions with a trained therapist in the dialysis facility. CBT sessions were conducted by a therapist with formal certification and prior clinical experience in this approach. CBT was administered chair-side while the participant was undergoing HD [35]. The study consisted of eight weekly, one-hour therapy sessions over a two-month period (Table 1).

**Table 1:** Cognitive behavioral therapy.

Session	Content of cognitive behavioral therapy session
1	Assess patient’s motivation for change, treatment goals, “stage of change”; evaluate need for patient to modify fluid intake and, comply with medical regimen.
2-3	Implement behavioral activation to increase participants’ enjoyable activities.
4-5	Educate participants on the relationship between dysfunctional Automatic thoughts and negative perception and Outcomes.
6	Teach and practice healthy living (compliance) skills in session.
7	Increase positive social contacts by initiating contact, building support network.
8	Plan for termination of therapy by identifying which interventions Were helpful and which were not, developing relapse prevention strategy.

#### 2.6.2. Emotion regulation therapy.

Thirty-two patients received eight sessions of ERT. Participants in the ERT program underwent eight individual, 60-minute therapy sessions with a trained therapist at the dialysis center [36]. ERT sessions were delivered by a therapist who possessed official certification and training in this therapeutic modality. Eight of these sessions happen weekly. The ERT sessions are conducted while the participant was receiving hemodialysis treatment (Table 2).

**Table 2:** Emotion regulation therapy.

Session	Content of emotion regulation therapy
1-2	Educate patients about emotions, including mechanisms and effects. This involves explaining the different types of emotions, their various dimensions, and the short and long-term consequences of experiencing them.
3	Evaluating the patient’s emotional state and their ability to manage their emotions.
4	Modify the circumstances that trigger the emotional response.
5	Attention shift.
6	Modify cognitive evaluations.
7	Modify the physical and behavioral reactions to emotions.
8	Identify and eliminate obstacles that hinder the implementation of the solution.

#### 2.6.3. Control group.

The control group received only the usual care provided by their primary healthcare service. Participants in the control group received routine medical care from their dialysis center, which included regular sessions with their primary nephrologist and healthcare team. Standard recommendations were provided by their physicians during follow up visits, covering areas such as nutrition, physical activity, complication prevention, medication adherence, and side effect management. No psychological interventions were administered to this group during the study period.

### 2.7. Statistical analysis

Following data collection, the descriptive statistics for the questionnaires were calculated using descriptive statistical methods, including means and standard deviations. Analysis of variance (ANOVA)was used to compare the sociodemographic characteristics of participants across the two intervention groups and the control group. The difference between pre- and post-intervention scores in both interventions and control groups was examined using t-tests and mixed analysis of covariance (MANCOVA) with repeated measures. Prior to conducting the statistical analysis, the normality of the data was examined through the utilization of the Shapiro-Wilk test, with a significance level set at 0.05. All analyses were performed using SPSS 26.0 software (IBM Corporation, Armonk, NY).

### 2.8. Ethical statement

This study was conducted in accordance with the principles of the Declaration of Helsinki and was approved by the Ethics Committee of Isfahan University of Medical Sciences (IR.MUI.MED.REC.1401.331). The study was also conducted based on the approved clinical trial protocol (Registration Number: IRCT20230119057155N1, First Registration: 16/02/2023, Access: https://irct.behdasht.gov.ir/trial/68123) and followed the CONSORT reporting guidelines. After coordination with the dialysis centers, written informed consent was obtained from all participants at the outset of the study.

## 3. Result

The findings of the present study were derived from the analysis of data obtained from 90 patients (n = 30 in ERT group n = 30 in CBT group n = 30 in control group). During the study, there was attrition, with six patients excluded from the ERT, CBT and control group, respectively. The clinical and demographic characteristics of the participants are presented in Table 3. There was no statistically significant difference in age, sex, marriage, BMI, dialysis onset, and education level among the ERT, CBT and control groups (P > 0.05) (Table 1). Although there was no statistically significant difference among the three groups regarding the number of dialysis sessions per week (P = 0.07), this variable was close to the threshold of significance. Therefore, it was considered a potential confounding factor and was statistically controlled using ANCOVA during the analysis of outcomes.

**Table 3:** Baseline characteristics of participants.

Variable		ERT group (n = 30)	CBT group (n = 30)	Control group (n = 30)	P
		Mean±SD	Mean±SD	Mean±SD	
Age	–	55.87 ± 13.47	56.80 ± 16.22	60.20 ± 17.62	0.455
BMI	–	25.83 ± 6.06	23.77 ± 4.02	24.33 ± 4.13	0.237
Dialysis onset	–	3.70 ± 2.98	3.60 ± 2.43	3.27 ± 2.33	0.796
Dialysis sessions	–	3.00 ± 0.26	2.80 ± 0.40	2.90 ± 0.30	0.07
Gender	Male	17	21	21	0.455
	Female	13	9	9	
Marital status	Married	20	23	26	0.187
	Single	10	7	4	
Education level	Diploma	15	14	15	0.956
	High Education	15	16	15	

The MANCOVA results indicated statistically significant differences between the CBT, ERT, and control groups on the combined adherence measures after controlling for baseline scores. Following the interventions, no significant difference in adherence was observed in the control group across the pre-test, post-test, and follow-up assessments; the rate of adherence decreased across all four subscales (dialysis, medication, fluid, and diet) and in total adherence. In the CBT group after treatment, adherence increased significantly after treatment; however, at follow-up, the dialysis adherence score decreased compared to the post-test while remaining above the baseline level. In the ERT group, adherence also increased post-treatment, but at follow-up the dialysis adherence score decreased compared to the post-test measurement.

The findings presented in Table 4 assess the changes in adherence scores among the three groups throughout the study. The data indicate that, prior to the intervention, adherence scores including total adherence, dialysis adherence, medication adherence, fluid adherence, and regimen adherence did not differ significantly among the groups. However, following the intervention, the ERT and CBT groups exhibited significantly higher adherence scores relative to the control group, with the most pronounced difference occurring between the CBT and control groups.

**Table 4:** The output of the ANCOVA model for comparing the effects of the study variables.

Groups	Before (Mean±SD)	After (Mean±SD)	Follow-Up (Mean±SD)	P Time	P Intervention	P Interaction
**Adherence total**						
ERT	8333.33 ± 210.53	920.00 ± 181.04	890.00 ± 155.30	0.004**	<0.001*	0.937
CBT	836.66 ± 192.95	1073.33 ± 89.28	1050.83 ± 93.88	<0.001*		
Control	911.66 ± 152.80	865.00 ± 158.33	835.00 ± 161.96	0.005*		
P	0.192	<0.001*	<0.001*			
**Adherence dialysis**						
ERT	456.66 ± 176.64	520.00 ± 153.18	483.33 ± 132.82	0.011**	<0.001*	0.165
CBT	490.00 ± 127.57	580.00 ± 48.42	574.16 ± 55.89	<0.001*		
Control	550.00 ± 125.43	526.66 ± 99.11	505.00 ± 96.80	0.075		
P	0.046**	0.006**	<0.001*			
**Adherence drug**						
ERT	126.66 ± 43.01	135.00 ± 41.83	133.33 ± 40.11	0.027**	<0.001*	0.48
CBT	121.66 ± 50.31	165.00 ± 43.84	155.00 ± 27.38	<0.001*		
Control	153.33 ± 61.49	145.00 ± 59.23	146.00 ± 58.62	0.123		
P	0.046**	<0.001*	<0.001*			
**Adherence liquid**						
ERT	120.00 ± 36.19	130.00 ± 28.16	131.66 ± 27.80	0.006*	<0.001*	0.487
CBT	95.00 ± 33.08	148.33 ± 15.99	145.00 ± 15.25	<0.001*		
Control	103.33 ± 29.16	96.66 ± 36.98	93.33 ± 36.51	0.175		
P	0.014**	<0.001*	<0.001*			
**Adherence regime**						
ERT	130.00 ± 44.72	135.00 ± 49.39	141.66 ± 43.71	0.143	<0.001*	0.208
CBR	130.00 ± 36.19	180.00 ± 28.16	176.66 ± 28.56	<0.001*		
Control	105.00 ± 57.75	96.66 ± 57.13	90.00 ± 60.74	0.081		
P	0.065	<0.001*	<0.001*			

*Significant P < 001, ** P < 0.05.

Furthermore, adherence scores across all categories increased over time in the ERT and CBT groups, while a decline was noted in the control group. The analysis reveals that the changes in adherence scores over time were statistically significant among the three groups, with the intervention groups exhibiting significant increases in scores, contrasted by a decrease in the control group (P < 0.001). These results suggest a positive effect of the interventions on adherence.

Post hoc analyses were conducted to delineate the nature of significant interactions and main effects, utilizing Tukey’s HSD test for group comparisons within time points and the Bonferroni correction for comparisons across time. The analysis revealed that the CBT group demonstrated significantly greater adherence than both the ERT and Control groups at both post-intervention and follow-up assessments (medication adherence post-intervention: CBT vs. ERT, P < 0.001; CBT vs. CONTROL, P < 0.001). The ERT group also showed a significant, though less consistent, advantage over the control group at follow up (adherence regime follow-up, P = 0.030) (S1 Table).

## 4. Discussion

ESRD is a life-threatening condition that necessitates dialysis or kidney transplant. Adherence to the treatment regimen is critical for these patients, as non-adherence adversely affects their health and quality of life [37]. This study compared the effect of ERT and CBT on improving adherence among dialysis patients. The findings indicated that both psychological interventions improved adherence behaviors. Consistent with previous research, the current findings underscore the efficacy of cognitive and behavioral strategies in enhancing adherence to prescribed dietary and fluid restrictions, medication, and dialysis treatment [38–40].

A randomized controlled trial conducted by Sharp et al. demonstrated that CBT reduced mean interdialytic weight gain in 56 hemodialysis patients. A significant difference was found between baseline and follow-up weight values in a long-term assessment (P < 0.001), indicative of enhanced adherence over time [41]. Cukor et al. similarly confirmed that 65 hemodialysis patients who received CBT showed significantly greater improvements in IDWG compared to a waitlist control group (P = 0.002) [35]. In our study, some participants subjectively reported that CBT sessions helped reduce their negative thoughts about dialysis and empowered them to take more responsibility for their health behaviors, which may have enhanced their treatment engagement. CBT’s effectiveness may be attributed to it is structured approach, which targets maladaptive beliefs and promotes adaptive coping strategies. By challenging dysfunctional cognitions and encouraging behavioral activation, CBT fosters greater self-efficacy and motivation for adherence. In contrast, while ERT also yielded significant results, its relatively narrower focus on emotional regulation processes such as cognitive reappraisal and expressive suppression might explain the smaller effect size observed in some adherence domains.

Patients with CKD, both those on dialysis and those who are not, frequently experience emotional distress, which can significantly impair their overall quality of life [42,43]. Previous research has suggested that non-adherence to medication regimens may be linked to cognitive decline and emotional or psychological problems [43,44]. Consequently, researchers have increasingly turned their attention to studying emotion regulation in chronic diseases, recognizing its potential impact on treatment non-adherence. Addressing these factors is crucial for effective adherence interventions. These results suggest that non-dialysis patients may employ more effective emotion regulation strategies, as they appear to be more proficient at re-evaluating their emotional responses and modulating their outward emotional expressions. In contrast, dialysis patients may struggle more with managing their emotions, resulting in lower scores in both cognitive reappraisal and expressive suppression. Supporting this, a study by Amar Ashan revealed that non-dialysis patients exhibited higher levels of emotion regulation, utilizing cognitive reappraisal and expressive suppression more frequently or effectively than dialysis patients [45]. In contrast, a study by Bazrafshan et al. in Iran found no significant correlation between treatment adherence and difficulty regulating emotions in dialysis patients. The authors attributed this lack of a significant relationship to the potential confounding effects of anxiety and depression, which could independently impact treatment adherence [37,46].

The observed effectiveness of CBT may be explained by it is comprehensive approach. By helping patients recognize and challenge maladaptive thought patterns that contribute to emotional distress and interfere with adherence behaviors, CBT can promote a more positive and motivated perspective toward dialysis. This, in turn, may enhance patients’ ability to manage their condition and adhere to treatment recommendations more consistently, although ERT also demonstrated beneficial effects, its focus on managing emotional responses may address a different, and perhaps narrower, set of barriers to adherence.

### 4.1. Limitations

The difficulty patients may have in understanding certain metaphors and analogies could be related to cultural factors. Therefore, the metaphors and narratives used in therapy need to should be reviewed and adapted to the Iranian cultural context to enhance their relevance and comprehensibility. Follow-up findings indicate that the effects of CBT on treatment adherence persisted beyond the active treatment phase. In some cases, the degree of improvement observed during the follow-up period even exceeded that of the initial post-treatment assessment. However, the duration of the follow up period was three months, which, while providing initial evidence of efficacy, may be insufficient to fully capture the long-term sustainability of adherence behaviors in a chronic condition such as ESRD. Future studies with extended follow up periods of six months to one year are warranted to confirm the durability of the intervention effects. Furthermore, future research on treatment adherence should incorporate objective or behavioral measures to enhance the validity of findings regarding the effectiveness of CBT and ERT. The use of such controlled measures as well as automatic recognition are [47] essential to ensure more rigorous and reliable conclusions about the impact of these therapeutic approaches.

## 5. Conclusion

Both CBT and ERT were effective in improving treatment adherence following an eight-session protocol. However, the results of this study suggest that CBT may yield relatively greater improvements in adherence compared to ERT for patients undergoing dialysis. Future investigations should employ longitudinal designs to assess the long-term durability of CBT and ERT effects on both treatment adherence and quality of life. Furthermore, exploring the specific cognitive and emotional mechanisms that mediate the impact of these therapies could provide deeper insight into how they ultimately influence adherence behaviors.

## Supporting information

S1 Table Results of Post Hoc test among the three groups. (DOCX)

S1 File Supporting information (2). (XLSX)

S2 File Scoring questions. (DOCX)

S3 File Study protocol. (DOCX)

S4 File CONSORT 2010. (DOCX)
