# Supplementary material for: Comparing the effectiveness of emotion regulation therapy and cognitive behavioral therapy on treatment adherence in hemodialysis patients: A randomized controlled clinical trial
Source: PLoS One. 2025 Dec 26;20(12):e0339162. doi: 10.1371/journal.pone.0339162 (PMC12742746; doi:10.1371/journal.pone.0339162)
Supplement: S1 Table — (DOCX) [file pone.0339162.s001.docx]

**Table S1. Results of Post Hoc test among the three groups**

| **Time** | **Groups** | **Mean Difference** | **Standard Error** | **P-value** | **Lower/Upper Bound** |
| --- | --- | --- | --- | --- | --- |
| Before Dialysis Adherence | ERT-CBT | -33.33 | 37.48 | 0.64 | [-122.70,56.04] |
|  | ERT-CONTROL | -93.33 | 37.48 | 0.03* | [-182.70, -3.96] |
|  | CBT-CONTROL | -60.00 | 37.48 | 0.25 | [-149.37,29.37] |
| Before Drug Adherence | ERT-CBT | 5.00 | 13.46 | 0.92 | [-27.11,37.11] |
|  | ERT-CONTROL | -26.66 | 13.46 | 0.12 | [-58.78,5.45] |
|  | CBT-CONTROL | -31.66 | 13.46 | 0.05 | [-63.78,0.45] |
| Before Liquid Adherence | ERT-CBT | 25.00 | 8.50 | 0.01** | [4.71,45.28] |
|  | ERT-CONTROL | 16.66 | 8.50 | 0.12 | [-3.61,36.94] |
|  | CBT-CONTROL | -8.33 | 8.50 | 0.59 | [-28.61,11.94] |
| Before Regime  Adherence | ERT-CBT | 0.00 | 12.15 | 1.00 | [-28.97,28.97] |
|  | ERT-CONTROL | 25.00 | 12.15 | 0.10 | [-3.97,53.97] |
|  | CBT-CONTROL | 25.00 | 12.15 | 0.10 | [-3.97,53.97] |
| Before Total  Adherence | ERT-CBT | -3.33 | 48.28 | 0.99 | [-118.46,111.79] |
|  | ERT-CONTROL | -78.33 | 48.28 | 0.24 | [-193.46,36.79] |
|  | CBT-CONTROL | -75.00 | 48.28 | 0.27 | [-190.12,40.12] |
| After Dialysis Adherence | ERT-CBT | -51.95 | 24.40 | 0.03* | [-100.49, -3.41] |
|  | ERT-CONTROL | 28.85 | 25.39 | 0.25 | [-21.64,79.36] |
|  | CBT-CONTROL | 80.81 | 24.85 | 0.01** | [-130.24, -31.37] |
| After Drug Adherence | ERT-CBT | -32.06 | 6.449 | 0.01** | [-44.88, -19.23] |
|  | ERT-CONTROL | 13.02 | 6.71 | 0.05* | [-0.32,26.37] |
|  | CBT-CONTROL | 45.08 | 6.56 | 0.01** | [-58.14, -32.02] |
| After Liquid  Adherence | ERT-CBT | -31.80 | 6.22 | 0.01** | [-44.18, -19.43] |
|  | ERT-CONTROL | 26.95 | 6.47 | 0.01** | [14.08,39.82] |
|  | CBT-CONTROL | 58.95 | 6.33 | 0.01** | [46.16,71.36] |
| After Regime  Adherence | ERT-CBT | -42.16 | 8.36 | 0.01** | [-58.79, -25.53] |
|  | ERT-CONTROL | 19.24 | 8.69 | 0.03* | [1.94,36.54] |
|  | CBT-CONTROL | 61.40 | 8.51 | 0.01** | [44.47,78.34] |
| After Total  Adherence | ERT-CBT | -151.57 | 28.63 | 0.01** | [-208.49, -94.64] |
|  | ERT-CONTROL | 96.43 | 29.06 | 0.01** | [38.65,154.20] |
|  | CBT-CONTROL | 248.00 | 29.02 | 0.01** | [190.29,305.70] |
| Follow-up Dialysis Adherence | ERT-CBT | -81.61 | 21.68 | 0.01** | [-124.74, -38.49] |
|  | ERT-CONTROL | 10.40 | 22.59 | 0.64 | [-34.46,55.27] |
|  | CBT-CONTROL | 92.02 | 22.08 | 0.01** | [48.10,135.94] |
| Follow-up Drug Adherence | ERT-CBT | -25.43 | 7.17 | 0.01** | [-39.70, -11.16] |
|  | ERT-CONTROL | 6.77 | 7.46 | 0.36 | [-8.07,21.62] |
|  | CBT-CONTROL | 32.20 | 7.30 | 0.01** | [17.67,46.74] |
| Follow-up  Liquid  Adherence | ERT-CBT | -26.41 | 6.36 | 0.01** | [-39.06, -13.76] |
|  | ERT-CONTROL | 29.31 | 6.61 | 0.01** | [16.15,42.47] |
|  | CBT-CONTROL | 55.72 | 6.47 | 0.01** | [42.84,68.61] |
| Follow-up Regime Adherence | ERT-CBT | -32.78 | 8.82 | 0.01** | [-50.34, -15.22] |
|  | ERT-CONTROL | 33.81 | 9.18 | 0.01** | [15.55,52.08] |
|  | CBT-CONTROL | 66.60 | 8.99 | 0.01** | [48.72,84.48] |
| Follow-up Total  Adherence | ERT-CBT | -159.20 | 27.70 | 0.01** | [-214.29, -104.12] |
|  | ERT-CONTROL | 93.52 | 28.12 | 0.01** | [37.34,149.16] |
|  | CBT-CONTROL | 252.45 | 28.09 | 0.01** | [196.61,308.29] |

Significance levels: *P ≤ 0.05, **P ≤ 0.01
