# Supplementary material for: Comparing the effectiveness of emotion regulation therapy and cognitive behavioral therapy on treatment adherence in hemodialysis patients: A randomized controlled clinical trial
Source: PLoS One. 2025 Dec 26;20(12):e0339162. doi: 10.1371/journal.pone.0339162 (PMC12742746; doi:10.1371/journal.pone.0339162)
Supplement: S2 File — (DOCX) [file pone.0339162.s003.docx]

**Scoring questions:**

| **Section Name** | **Question Number** | **Targeted Area in The Item** | **To Record Value of (Points)** |
| --- | --- | --- | --- |
| Section 1: General Information (5 items) | 1, 2, and 3 | Fact related to previous RRT history | No value |
|  | 4 and 5 | Fact related to transportation situation to get HD | No value |
| Section 2: HD Treatment (14  items) | 6 and 7 | Fact related to HD schedule | No value |
|  | 8 | Perception of patients on HD schedule | No value |
|  | 9 and 10 | Information about counseling on HD | No value |
|  | 11 | Perception on importance of HD adherence | No value  Analyze responses using descriptive statistics |
|  | 12 | Understanding level on importance of HD | No value  Analyze responses using descriptive statistics |
|  | 13 | Perception of patients on HD | No value |
|  | 14 | Frequency of missing HD during last month | Response category 1→300 Response category 2→200 Response category 3→100 Response category 4→50 Response category 5→0 |
|  | 15 | Reason for missing HD | No value (Note: If patients missed HD due to medical reasons (if the answer is 4, 6, or 7), adjust scores from question number 14 and give a full credit (300 points) |
|  | 16 | Supplementary question for Question 15 (psychophysical symptoms) | No value |
|  | 17 | Frequency of shortening HD during last month | Response category 1→200 Response category 2→150 Response category 3→100 Response category 4→50 Response category 5→0 |
|  | 18 | Duration of shortening HD during last month | Response category 1→100 Response category 2→75 Response category 3→50 Response category 4→25 Response category 5→0 |
|  | 19 | Reason for shortening HD treatment | No value (Note: If patients shortened HD due to medical reasons (if the answer is 2, 5, 6 or 11), adjust scores from question number 17 & 18 and give a full credit (200 and 100 points) |
| Section 3: Medication (9 items) | 20 and 21 | Information about counseling on medication | No value |
|  | 22 | Perception on importance of medication adherence | No value Analyze responses using descriptive statistics |
|  | 23 | Understanding level on importance of medication | No value.  Analyze responses using descriptive statistics |
|  | 24 and 25 | Fact related to difficulty with taking medicines | No value |
|  | 26 | Frequency of missing medication during last month | Response category 1→200 Response category 2→150 Response category 3→100  Response category 4→50 Response category 5→0 |
|  | 27 | Reason for missing medication | No value (Note: If patients missed medication due to medical reasons (if the answer is 6 or 7) adjust scores from the question number 26 and give a full credit (200 points) |
|  | 28 | Supplementary question for Question 27 (psychophysical symptoms) | No value |
| Section 4: Fluid Restriction (10 items) | 29 and 30 | Information about counseling on fluid restriction | No value |
|  | 31 | Fluid restriction: Self-monitoring (Frequency) | Response category 1→200 Response category 2→150 Response category 3→100 Response category 4→50 Response category 5→0 |
|  | 32 | Perception on importance of fluid restriction | No value  Analyze responses using descriptive statistics |
|  | 33 | Understanding level on importance of fluid restriction | No value Analyze responses using descriptive statistics |
|  | 34 and 35 | Fact related difficulty with limiting fluid intake | No value |
|  | 36 | Types of difficulty following fluid restriction (additional question to #35) | No value |
|  | 37 and 38 | Information on weighing at home (not mandatory requirements for all ESRD patients) | No value |
| Section 5: Dietary Restriction (8 items) | 39 and 40 | Information about counseling on dietary recommendations | No value |
|  | 41 | Perception on importance of dietary recommendations | No value  Analyze responses using descriptive statistics |
|  | 42 | Understanding level on importance of dietary recommendations | No value  Analyze responses using descriptive statistics |
|  | 43 and 44 | Fact related to difficulty with following dietary recommendations | No value |
|  | 45 | Types of difficulty following fluid restriction (Additional question to #44) | No value |
|  | 46 | Dietary restriction: Self-monitoring (Frequency) | Response category 1→200 Response category 2→150 Response category 3→100 Response category 4→50 Response category 5→0 |
