# Supplementary material for: Comparing the effectiveness of emotion regulation therapy and cognitive behavioral therapy on treatment adherence in hemodialysis patients: A randomized controlled clinical trial
Source: PLoS One. 2025 Dec 26;20(12):e0339162. doi: 10.1371/journal.pone.0339162 (PMC12742746; doi:10.1371/journal.pone.0339162)
Supplement: S3 File — (DOCX) [file pone.0339162.s004.docx]

**Title**

Comparing the Effectiveness of Emotion Regulation Therapy and Cognitive Behavioral Therapy on Treatment Adherence in Hemodialysis Patients: A Randomized Controlled Clinical Trial

**Names protocol contributors**

Seyedeh Hanieh Salimi Arshad Moghaddam Pishkhani, Mohammad Javad Tarrahi, Fatemeh Zargar

**Abstract**

**Background:** Non-adherence is a common challenge among patients undergoing hemodialysis (HD) in the dialysis community. This randomized controlled trial compared the efficacy of Emotion Regulation Therapy (ERT) and Cognitive Behavioral Therapy (CBT) on improving treatment adherence in hemodialysis patients, with a control group receiving standard care.

**Methods:** Ninety hemodialysis patients were equally randomized into ERT, CBT, and control groups (n=30 each), with 6 attrition cases per group. Baseline demographics (age, BMI, dialysis duration, education) showed no significant intergroup differences (p>0.05). Adherence was assessed across five domains: dialysis, medication, fluid intake, dietary regimen, and total adherence. A MANCOVA/ANCOVA model analyzed changes at pre-intervention, post-intervention, and 3-month follow-up, controlling for baseline characteristics.

**Results:** Both intervention groups demonstrated significant adherence improvements post-treatment versus controls (P<0.001). CBT showed superior immediate effects, with total adherence scores increasing from 836.66±192.95 to 1073.33±89.28 (Δ+28.3%), while ERT improved from 833.33±210.53 to 920.00±181.04 (Δ+10.4%). At follow-up, CBT maintained higher adherence (1050.83±93.88 vs. ERT's 890.00±155.30), though both groups experienced dialysis adherence declines from post-treatment peaks (CBT: 580→574.16; ERT: 520→483.33). Control group adherence deteriorated across all domains (total: 911.66→835.00). Time-intervention interactions were significant for total adherence (P<0.001), dialysis (P=0.006), and medication adherence (p<0.001), with largest CBT effects on fluid restriction adherence (Δ+56.1% vs. ERT's Δ+8.3%).

**Discussion:** While both therapies enhanced adherence, CBT produced greater short-term improvements, particularly in behavioral domains (fluid/dietary compliance), whereas ERT showed better maintenance of medication adherence. The differential trajectory patterns suggest CBT's structured behavioral strategies may offer immediate benefits, while ERT's emotion-focused techniques could support longer-term regimen acceptance. Integration of both approaches into renal care protocols may optimize adherence outcomes.

**Trial registration:** IRCT20230119057155N1, **First Registration:** 16/02/2023**,** <https://irct.behdasht.gov.ir/trial/68123>

**Keywords**

Emotion Regulation Therapy, Cognitive Behavioral Therapy, Adherence, Dialysis, randomized controlled trial.

**Administrative information:**

*Trials* guidance: please include this text in your protocol just above the Administrative information table:

Note: the numbers in curly brackets in this protocol refer to SPIRIT checklist item numbers. The order of the items has been modified to group similar items (see <http://www.equator-network.org/reporting-guidelines/spirit-2013-statement-defining-standard-protocol-items-for-clinical-trials/>).

| **Title {1}** | Comparing the Effectiveness of Emotion Regulation Therapy and Cognitive Behavioral Therapy on Treatment Adherence in Hemodialysis Patients: A Randomized Controlled Clinical Trial |
| --- | --- |
| **Trial registration {2a and 2b}.** | **IRCT20230119057155N1, First Registration: 16/02/2023,** [**https://irct.behdasht.gov.ir/trial/68123**](https://irct.behdasht.gov.ir/trial/68123) |
| **Protocol version {3}** | Version 1 |
| **Funding {4}** | This study was financially supported by the Isfahan University of Medical Sciences, Isfahan, Iran ((grant number:3401480). |
| **Author details {5a}** | **1.** **Seyedeh Hanieh Salimi Arshad Moghaddam Pishkhani**  *Department of Health psychology, School of Medicine, Isfahan University of Medical Sciences, Isfahan, Iran*  ***ORCID:*** *0009-0004-4002-5242*  ***Email:*** *haniehsalimi98@gmail.com*  **2.** **Mohammad Javad Tarrahi**  *Professor of Epidemiology Department of Epidemiology and Biostatistics, School of Health, Isfahan University of Medical Sciences, Isfahan, Iran*  ***ORCID:*** *0000-0001-7875-4572*  ***Email:*** *MJ.TARRAHI@gmail.com*  **3.** **Fatemeh Zargar**  Associated Professor, Department of Health Psychology, Isfahan University of Medical Sciences, Isfahan, Iran  **ORCID:** 0000-0003-2121-3323  **Email:** fatemehzargar@gmail.com  ***Corresponding Author:**  Fatemeh Zargar  Associated Professor, Department of Health Psychology, Isfahan University of Medical Sciences, Isfahan, Iran.  Postal code: 81777-46674; Tel: +98 31 37928109; Fax: +98 31 37928108; Email: fatemehzargar@gmail.com |
| **Name and contact information for the trial sponsor {5b}** | Isfahan University of Medical Sciences Research Committee. |
| **Role of sponsor {5c}** | Issuance of approval and financing for project implementation. |

**Introduction**

**Background and rationale {6a}**

This study aims to compare two treatment approaches, emotion regulation therapy and cognitive behavioral therapy, on adherence for patients with chronic kidney disease. The specific goal of this study was to evaluate the effectiveness of emotion regulation therapy in improving treatment adherence compared to cognitive behavioral therapy.

**Objectives {7}**

1. A comparison of the mean treatment adherence scores among renal and hemodialysis patients in the emotion regulation treatment group at three different time points: prior to the intervention, immediately following the intervention, and three months post-intervention.
2. A comparison of the mean treatment adherence scores among renal and hemodialysis patients in the cognitive behavioral group at three different time points: prior to the intervention, immediately following the intervention, and three months post-intervention.

**Trial design {8}**

The present study is a parallel type with experimental methods of pre-test, post-test, and follow up with a control group. This number of samples was taken from people who referred to the hemodialysis department to receive dialysis hospitals. Randomization of this study with the method of simple randomization.

**Methods: Participants, interventions and outcomes**

**Study setting {9}**

This clinical trial study was conducted between February, 20,2023 and April,17,2024. Ninety-six hemodialysis patients enrolled to 8 session of cognitive behavioral therapy, emotion regulation therapy and control group. Randomization of this study with the method of simple randomization. For this purpose, an online website (www.rresearchrandomizer.com) was used, and finally a list was determined in which the letter in front of each number indicates which group the person belongs to.

**Eligibility criteria {10}**

The inclusion criteria for the selection of patients were: 1) Being between 20 and 80 years old; 2) Referral by a nephrologist with the diagnosis of the need for dialysis; 3) participants should have a minimum level of education; 4) Filling the informed consent form; 5) Not suffering from a serious psychiatric illness such as major depression, bipolar, psychotic disorders, etc. based on a short diagnostic interview; 6) Absence of substance abuse or addiction; 7) Failure to participate in other psychotherapy programs from 6 months ago until now.

The exclusion criteria for the selection of patients were: 1) Absence of more than two treatment sessions; 2) Occurrence of severe physical problems; 3) Hospitalization; 4) Failure to complete the questionnaire.

**Who will take informed consent? {26a}**

Seyedeh Hanieh Salimi Arshad Moghaddam Pishkhani as a researcher

**Additional consent provisions for collection and use of participant data and biological specimens {26b}**

Not applicable in this study.

**Interventions**

**Explanation for the choice of comparators {6b}**

Not applicable in this study.

**Intervention description {11a}**

Thirty-two patients received 8 sessions of cognitive behavioral therapy. Participants in the CBT undergo eight 60-minute individual sessions with a trained therapist in the dialysis facility (8 weekly sessions).

Thirty-two patients received 8 sessions of emotion regulation therapy. people in the ERT program receive eight individual , 60-minute therapy sessions with a trained therapist at the dialysis center ^30^. Eight of these sessions happen weekly. The control group only received the usual care from their primary healthcare service.

**Criteria for discontinuing or modifying allocated interventions {11b}**

The exclusion criteria for the selection of patients were: 1) Absence of more than two treatment sessions; 2) Occurrence of severe physical problems; 3) Hospitalization; 4) Failure to complete the questionnaire.

**Strategies to improve adherence to interventions {11c}**

Not applicable in this study.

**Relevant concomitant care permitted or prohibited during the trial {11d}**

use of medications that could interfere with the treatment.

**Provisions for post-trial care {30}**

Not applicable in this study.

**Outcomes {12}**

While both therapies enhanced adherence, CBT produced greater short-term improvements, particularly in behavioral domains (fluid/dietary compliance), whereas ERT showed better maintenance of medication adherence. The differential trajectory patterns suggest CBT's structured behavioral strategies may offer immediate benefits, while ERT's emotion-focused techniques could support longer-term regimen acceptance. Integration of both approaches into renal care protocols may optimize adherence outcomes.

**Participant timeline {13}**


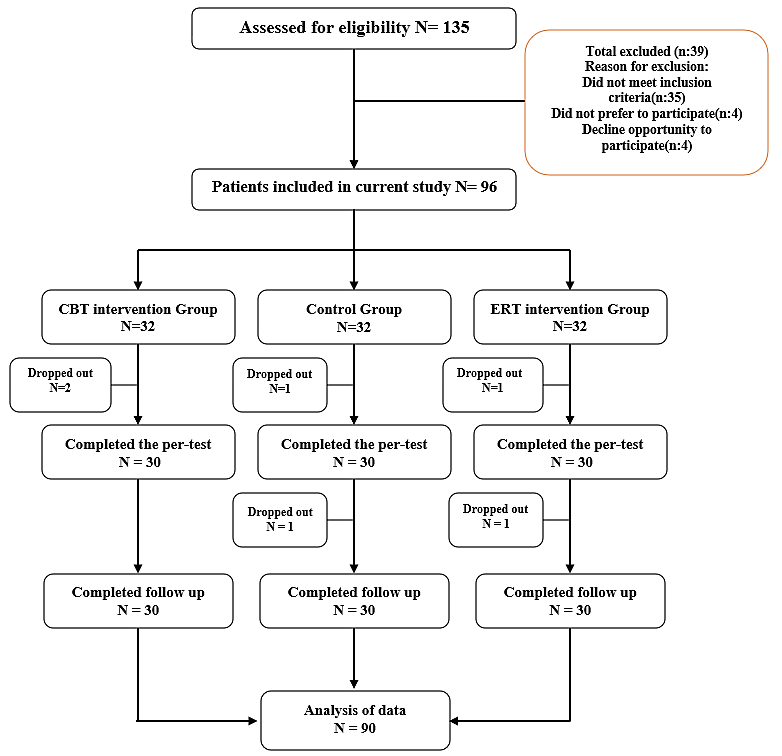


**Sample size {14}**

This number of samples was taken from people who referred to the hemodialysis department to receive dialysis hospitals. The sample size for each experimental group was calculated using the following the equation (1):

Equation (1): $n={(\lambda}_{g,\alpha,1-\beta})/\Delta$ and $\Delta=\frac{1}{\sigma^{2}} \sum_{i=1}^{k} {(\mu-\bar{\mu})}^{2}$

The parameters for this calculation were defined as follows:

- Number of groups (*k*) = 3, Type I error (α*α*) = 0.05, Type II error (*β*) = 0.2 (power = 80%)
- Group means: μ1=900, μ2=970, μ3=860
- Pooled standard deviation (*σ*) = 130.26 (derived from adherence score variance) ^26^
- Grand mean (*μ*ˉ​) = 910 (calculated as $\frac{1}{3}\sum_{i=1}^{3} \mu_{i}$)

Substituting these values into the equation yielded a required sample size of 30 participants per group. Consequently, the total sample size for the study was determined to be 90 participants, ensuring adequate statistical power to detect intergroup differences in adherence scores under the specified error thresholds.

**Recruitment {15}**

SPIRIT guidance: Strategies for achieving adequate participant enrolment to reach target sample size.

**Assignment of interventions: allocation**

**Sequence generation {16a}**

Not applicable in this study.

**Concealment mechanism {16b}**

Not applicable in this study.

**Implementation {16c}**

Seyedeh Hanieh Salimi Arshad Moghaddam Pishkhani as a researcher will generate the allocation sequence, he will enrol participants, and he will assign participants to interventions.

**Assignment of interventions: Blinding**

**Who will be blinded {17a}**

The person administering the treatment is not aware of the outcome variables, and the person in charge of data analysis is not aware of the intervention groups.

**Procedure for unblinding if needed {17b}**

Not applicable in this study.

**Data collection and management**

**Plans for assessment and collection of outcomes {18a}**

Participant enrollment started on the February, 20,2023, and trial completed on the April,17,2024, at different hospitals in Iran. Patients were approached in the hemodialysis center and asked to undergo an assessment to determine eligibility for the clinical trial. Following baseline assessment, eligible participants were randomly assigned to one of three groups: emotion regulation therapy g, cognitive behavioral therapy, and control group. Pre-intervention assessments were performed Patients before, the two intervention groups received individual therapy for two months. After 2month, post-intervention assessments were performed and after 3 month follow up were performed.

**Plans to promote participant retention and complete follow-up {18b}**

In the first session of the intervention, the benefits of participating in the course were explained to the participants.

**Data management {19}**

SPIRIT guidance: Plans for data entry, coding, security, and storage, including any related processes to promote data quality (eg, double data entry; range checks for data values). Reference to where details of data management procedures can be found, if not in the protocol.

**Confidentiality {27}**

All information collected in this research is confidential and has not been shared with any individual or group. In addition, this has been explained to the participants in the informed consent form.

**Plans for collection, laboratory evaluation and storage of biological specimens for genetic or molecular analysis in this trial/future use {33}**

Not applicable in this study.

**Statistical methods**

After collecting the data, descriptive statistics of the questionnaires were determined using descriptive statistical methods such as mean and standard deviation. ANOVA analysis were used to compare the sociodemographic of participants in both the interventions and control groups. The difference between pre- and post-intervention scores in both interventions and control groups was examined using t-tests and mixed analysis of covariance (MANCOVA) with repeated measures. Also, prior to conducting the statistical analysis, the normality of the data was examined through the utilization of the Shapiro-Wilk test, with a significance level set at 0.05. All analyses were performed using SPSS 26.0 software (IBM Corporation, Armonk, NY).

**Interim analyses {21b}**

SPIRIT guidance: Description of any interim analyses and stopping guidelines, including who will have access to these interim results and make the final decision to terminate the trial.

**Methods for additional analyses (e.g. subgroup analyses) {20b}**

SPIRIT guidance: Methods for any additional analyses (eg, subgroup and adjusted analyses).

**Methods in analysis to handle protocol non-adherence and any statistical methods to handle missing data {20c}**

SPIRIT guidance: Definition of analysis population relating to protocol non-adherence (eg, as randomised analysis), and any statistical methods to handle missing data (eg, multiple imputation).

**Plans to give access to the full protocol, participant level-data and statistical code {31c}**

SPIRIT guidance: Plans, if any, for granting public access to the full protocol, participant-level dataset, and statistical code.

**Oversight and monitoring**

**Composition of the coordinating centre and trial steering committee {5d}**

Trials guidance: Provide information on the composition, roles and responsibilities of the coordinating centre and trial steering committee and all groups providing day to day support for the trial. There will always be a group running the trial day-to-day and providing organisational support and knowing how often they will meet, plus information on other committees providing oversight such as a Trial Steering Committee, and how often they will meet over the course of the trial, is what we need for item 5d. We do not need names of staff.

SPIRIT guidance: Composition, roles, and responsibilities of the coordinating centre, steering committee, endpoint adjudication committee, data management team, and other individuals or groups overseeing the trial, if applicable (see Item 21a for data monitoring committee).

**Composition of the data monitoring committee, its role and reporting structure {21a}**

The Research Committee of Isfahan University of Medical Sciences has fully supervised the implementation of this intervention and an approval code has been issued by this committee.

**Adverse event reporting and harms {22}**

In this study, no danger threatens the participants.

**Frequency and plans for auditing trial conduct {23}**

Not applicable in this study.

**Plans for communicating important protocol amendments to relevant parties (e.g. trial participants, ethical committees) {25}**

SPIRIT guidance: Plans for communicating important protocol modifications (eg, changes to eligibility criteria, outcomes, analyses) to relevant parties (eg, investigators, REC/IRBs, trial participants, trial registries, journals, regulators).

**Dissemination plans {31a}**

SPIRIT guidance: Plans for investigators and sponsor to communicate trial results to participants, healthcare professionals, the public, and other relevant groups (eg, via publication, reporting in results databases, or other data sharing arrangements), including any publication restrictions.

**Discussion**

*Trials* guidance: This should include a discussion of any practical or operational issues involved in performing the study and any issues not covered in other sections.

**Trial status**

**Approval date** 16/02/2023

**Abbreviations**

*Trials* guidance: If abbreviations are used in the text they should be defined in the text at first use, and a list of abbreviations should be provided.

**Declarations**

The authors declared no potential conflicts of interest with respect to the research, authorship, and/or publication of this article.

**Acknowledgements**

All authors thank the Vice-Chancellor of Research and Technology of Isfahan University of Medical Sciences, as well as all caregivers, psychologists and people who participated in this study.

**Authors’ contributions {31b}**

**Seyedeh Hanieh Salimi Arshad Moghaddam Pishkhani**: Investigation, Methodology, Writing – original draft, Resources. **Mohammad Javad Tarrahi:** Writing – review & editing, Software. **Fatemeh Zargar:** Conceptualization, Writing – review & editing, Supervision, Project administration, Validation, Visualization**.**

**Funding {4}**

This study was financially supported by the Isfahan University of Medical Sciences, Isfahan, Iran (grant number: 3401480).

**Availability of data and materials {29}**

The datasets generated and/or analyzed during the study are not publicly available and the authors can provide the data upon reasonable request.

**Ethics approval and consent to participate {24}**

This study was approved by the Ethics Committee of Isfahan University of Medical Sciences (IR.MUI.MED.REC.1401.331). After coordinating with nursing homes, written informed consent was obtained from all participants at the beginning of the study.

**Consent for publication {32}**

**Informed Consent Form**

The Department of Psychology at Isfahan University of Medical Sciences supports the practice of protection of human participants in research.  The following will provide you with information about the experiment that will help you in deciding whether or not you wish to participate.  If you agree to participate, please be aware that you are free to withdraw at any point throughout the duration of the experiment without any penalty.

In this study we will ask you to participate in a therapeutic intervention that will probably increase adherence to the dialysis process.

If you have any Problem, please inform the experimenter and the study will end now.  All information you provide will remain confidential and will not be associated with your name.  If for any reason during this study you do not feel comfortable, you may leave the laboratory and receive credit for the time you participated and your information will be discarded.

Your participation in this study will require approximately 2 months minutes.  When this study is complete you will be provided with the results of the experiment if you request them, and you will be free to ask any questions.

If you have any further questions concerning this study please feel free to contact us through phone or email: …………….. (+98………….).

Please indicate with your signature on the space below that you understand your rights and agree to participate in the experiment.

Your participation is solicited, yet strictly voluntary. All information will be kept confidential and your name will not be associated with any research findings.
 
______________________________                                    ______________________________
            Signature of Participant                                                 NAME, Investigator

**Competing interests {28}**

SPIRIT guidance: Financial and other competing interests for principal investigators for the overall trial and each study site.

*Trials* guidance: All financial and non-financial competing interests must be declared in this section. See our [editorial policies](https://www.biomedcentral.com/getpublished/editorial-policies#availability+of+data+and+materials) for a full explanation of competing interests. If you are unsure whether you or any of your co-authors have a competing interest please contact the editorial office. Please use the authors initials to refer to each authors' competing interests in this section. If you do not have any competing interests, please state: "The authors declare that they have no competing interests" in this section.

**Authors’ information (optional)**

*Trials* guidance: This section is optional.

You may choose to use this section to include any relevant information about the author(s) that may aid the reader's interpretation of the article, and understand the standpoint of the author(s). This may include details about the authors' qualifications, current positions they hold at institutions or societies, or any other relevant background information. Please refer to authors using their initials. Note this section should not be used to describe any competing interests.
